# Management of Skull-Base Meningiomas With Extracranial Extensions: Clinical Features, Radiological Findings, Surgical Strategies, and Long-Term Outcomes

**DOI:** 10.3389/fneur.2022.855973

**Published:** 2022-07-01

**Authors:** Wenbo He, Zhiyong Liu, Danyang Jie, Liansha Tang, Haibo Teng, Jianguo Xu

**Affiliations:** ^1^Department of Neurosurgery, West China Hospital of Sichuan University, Chengdu, China; ^2^West China School of Medicine, Sichuan University, Chengdu, China; ^3^Department of Biotherapy, Cancer Center, West China Hospital of Sichuan University, Chengdu, China

**Keywords:** meningioma, extracranial extension, surgical resection, prognosis, radiological findings

## Abstract

**Objectives:**

The aim of this research was to summarize the clinical and prognostic features of the skull-base meningiomas with extracranial extensions, and enhance the management of skull-base communicative meningiomas.

**Methods:**

We retrospectively studied the medical records and analyzed the follow-up information of 53 patients who have done surgery for skull-base meningiomas with extracranial extensions in West China Hospital of Sichuan University from 2009 to 2020.

**Results:**

The incidence of skull-base meningiomas with extracranial extensions was 0.74%. The average diagnosis age was 45.9 years, with a 1:3.1 men to women ratio. WHO grade I was seen in 84.9% of patients, and higher grades were found in 15.1%. Heterogeneous enhancement, high bone invasion rate, high incidence of peritumoral edema, and high dural tail sign rate were typical imaging features. Routine craniotomy and endoscopic endonasal approach were adopted, and gross total resection was performed in 62.3% of cases with 20.8% postoperative complication rates. The average follow-up time was 61.5 months, with a recurrence rate of 34.9%. By survival analysis, the extent of resection (*p* = 0.009) and the histological grade (*p* = 0.007) were significantly related to the prognosis. Adjuvant radiotherapy proved beneficial in patients with subtotal resection (*p* = 0.010) and high-grade meningiomas (*p* = 0.018).

**Conclusions:**

Skull-base meningiomas with extracranial extensions were sporadic. According to the tumor location and communication way showed by the preoperative imaging, routine craniotomy or endoscopic endonasal approach with a reasonable skull-base repair strategy could be adopted to achieve the maximum tumor resection. Maximized resection, adjuvant radiotherapy, and low histological grade indicate a better prognosis.

## Introduction

Meningiomas have been the most common central nervous system (CNS) tumors, accounting for 37.6% of CNS tumors (1). In contrast, the internal and external communication of the skull-base meningioma is a rare type of meningiomas (2). This kind of meningiomas passes through natural cavities such as the optic foramen, superior orbital fissure or destroy bone to achieve intracranial and extracranial links and involve sellar area, supraorbital fissure, orbit tips, and other critical structural areas rich in blood vessels and nerves. Due to the rarity of skull-base communicative meningiomas, there is no established management method. In our cohort, we retrospectively reviewed the communication way, clinical symptoms, radiological characteristics, treatment strategies, and prognosis of 53 patients with skull-base meningiomas with extracranial extensions operated in West China Hospital of Sichuan University (about 2,800 CNS tumors operations are performed per year), to summarize the clinical and prognostic features and enhance the management of skull-base communicative meningiomas.

## Methods

### Patient Population

We retrospectively analyzed the medical record and radiological information of skull-base communicative meningiomas at West China Hospital of Sichuan University, from January 2009 to May 2020. The informed consent of these patients was approved by the West-China Hospital Research Ethics Committee. The medical record and the image data were extracted from the hospital information system and the hospital picture archiving and communication systems (PACS), retrospectively. MRI data were acquired by magnetic resonance plain scan, enhanced scan, and intraoperative navigation. Two skilled radiologists extracted separately the radiological imaging features of these meningiomas from the preoperative MRI combined with CT images. Inclusion criteria include: precise pathological diagnosis, skull-base meningiomas with extracranial extensions, and underwent microsurgery as a primary treatment. Exclusion criteria include: recurring or metastatic tumor, pathological diagnosis combined with other lesions, and have received radiotherapy. The diagnosis was verified by histopathological evaluation including immunohistochemical analysis, and the meningiomas were classified according to the WHO Classification of Tumors of the Central Nervous System (WHO 2021 5th version).

### Surgical Strategy

All patients included in the study underdone surgery by routine craniotomy or endoscopic endonasal approach in our center. For patients with internal carotid artery involvement identified on preoperative MRI, computed tomographic angiography (CTA) was taken to determine tumor impact on the internal carotid artery. For patients with suspected internal carotid artery compression, MR angiography (MRA)/digital subtraction angiography (DSA), and internal carotid artery balloon occlusion test were performed to evaluate the degree of compression. The surgical positions were all in the supine position. According to the location of the main body of the tumor, orbitofrontal approach, pterional approach, orbital zygomatic approach, and endoscopic endonasal approach were taken to deal with the communicative lesions. Simpson resection grade standard was applied to evaluate the extent of resection (EOR) (3). Levels I and II were defined as gross total resections (GTRs), while levels III and IV were subtotal resections. The postoperative skull-base reconstruction strategy was flexible. For intact dura mater and skull-base bone defect less than 3 cm, no repair will be made. For defect ≥ 3 cm and dura mater intact, use pedicled temporalis or frontal muscle galette aponeurosis to reconstruct the skull base by layer flip, then fix and rebuild with titanium mesh. For significant tissue defects and dural defects, first, fill the dura mater with autologous adipose tissue, then use temporal muscle fascia, pedicled skull periosteum or artificial dural to repair the dura mater, and finally reconstruct the skull base by the above method.

### Follow-Up Data

Ten patients were lost to follow-up on discharge and the other 43 patients received a regular telephone or outpatient follow-up after discharge. Enhanced MRI scans for these patients were required to be performed 3 months after surgery and then once a year after that. Tumor recurrence rate combined with disease-free survival (DFS) was applied to evaluate the prognosis. DFS was defined as the time from surgery to tumor recurrence or death from and cause. In addition, we evaluated the Karnofsky performance status (KPS) at the last follow-up of these patients to compare the long-term outcomes.

### Statistical Methods

All statistical analyses were performed by SPSS (version 22.0, IBM). To assess predictors of DFS, we included the following factors: age, sex, communication way, WHO grade, the EOR, and adjuvant radiotherapy. K-M curve was adopted to describe the DFS, and a log-rank test was conducted to compare the difference between the curves, while the Cox proportional hazards model was conducted to do multivariate analysis. Besides, a *P* value less than 0.05 was considered statistically significant.

## Results

### Incidence, Age, Sex, and Histopathology

From January 2009 to May 2020, 7,202 patients with meningiomas underwent surgery at the Department of Neurosurgery, West-China Hospital of Sichuan University. Of these patients, only 60 were skull-base meningiomas with extracranial extensions. We excluded seven recurring meningiomas, and the final number of patients with communicative meningiomas was 53 (0.74%). The features of the study population were listed in Table 1. The average diagnosis age was 45.9 years (ranging from 2 to 72) with a 1:3.1 men to women ratio. Concerning histopathology, WHO grade I was confirmed in 45 patients (84.9%), WHO grade II was confirmed in 8 patients (15.1%), and no WHO grade III was reported in our series. Further pathological examination was done in 22 patients. Some potentially prognostic indicators such as Ki-67 and progesterone receptor (PR) were added to **Table 4**.

**Table 1:** Characteristics of study population.

**Category**	**Value**
**Gender**	
Male	13 (24.5%)
Female	40 (75.5%)
**Age**	
<50	27 (50.9%)
≥50	26 (49.1%)
**Extracranial extensions**	
Orbit	45 (84.9%)
Nasal cavity or paranasal sinus	9 (17.0%)
Infratemporal or pterygopalatine fossa	3 (5.7%)
**Histological grade**	
Grade 1	45 (84.9%)
Grade 2	8 (15.1%)
Grade 3	0

### Communication Ways and Clinical Symptoms

A total of 84.9% of the lesions were communicated to the orbit, 17.0% of the lesions were communicated to the nasal cavity, and 5.7% of the lesions were communicated to the infratemporal or pterygopalatine fossa. In addition, 43.4% of the lesions communicated through the natural cavity, while 56.6% of lesions communicated through the destruction of bone. The period from initial symptoms to the surgical intervention ranged between 0.47 and 144.0 months, with a median of 12.0 months and an average of 28.7 months. The most frequent symptoms were related to visual impairment in 40 patients (75.5%), ten of which were completely blind, followed by headache or eye pain in 28 patients (53.9%), progressive exophthalmos in 26 patients (49.1%) and the average degree of exophthalmos is 9.17 mm, the disorder of ocular movement in 8 patients (15.1%), dysosmia in 3 patients (5.7%), and epilepsy in 2 patients (3.8%). Detailed clinical manifestations were summarized in Table 2. In addition, the lesions of three patients (5.7%) without any symptoms were found during physical examination.

**Table 2:** Different clinical manifestations of the patients.

**Clinical manifestations**	**Number of patients**
Visual impairment	40 (75.5%)
Headache or eye pain	28 (52.8%)
Progressive exophthalmos	26 (49.1%)
Disorder of ocular movement	8 (15.1%)
Dysosmia	3 (5.7%)
Epilepsy	2 (3.8%)
Facial prominence	2 (3.8%)
Facial numbness	1 (1.9%)
Physical examination	3 (5.7%)

### Radiological Findings

In our series, T1, T2 weighted, and enhancement images could be obtained in 38 patients. Based on radiological imaging, most tumors present irregular shape (60.5%) and clear tumor-brain boundary (81.6%). With regard to tumor size, the maximum diameter of the meningiomas ranged from 10 to 91 mm, with a mean of 45.90 (±20.9) mm. Peritumoral edema and cystic degeneration were respectively found in 14 cases and 2 cases. Skull imaging was abnormal in 21 cases, including hyperostosis and destructive absorption of the cranial bone. Detailed radiological features were summarized in Table 3.

**Table 3:** Radiological features of available 38 patients.

**Characteristics**	**Number of cases**
Shape	Regular	15 (39.5%)
	Irregular	23 (60.5%)
Boundary	Clear	31 (81.6%)
	Unclear	7 (18.4%)
Cystic/Calcification	Presence	9 (23.7%)
	Absence	29 (76.3%)
Dural tail sign	Presence	31 (81.6%)
	Absence	7 (18.4%)
Skull invasion	Presence	21 (55.3%)
	Absence	17 (44.7%)
Peritumoral edema	Presence	14 (36.8%)
	Absence	24 (63.2%)
Tumor enhancement	Homogeneous	17 (44.7%)
	Heterogeneous	21 (55.3%)

### Surgical Strategies and Outcome

According to the location of meningiomas, 16 cases were used with the orbitofrontal approach, 15 cases with the pterional approach, 19 cases with the orbital zygomatic approach, and 3 cases with the endoscopic endonasal approach. GTR (Simpson grades I and II) has been completed in 33 cases (62.3%), while the remaining patients have undergone subtotal tumor resection (Simpson grade III or IV). Regarding the defect of the skull base, postoperative skull-base reconstruction was carried out in 31 patients (58.5%). Postoperative adjuvant radiotherapy was done in 13 patients (24.5%) to deal with the remnant after subtotal resection (STR) or high-grade meningiomas.

There was no postoperative mortality, and the postoperative complication rate was low (*n* = 11, 20.8%). The infection was the most common postoperative complication during hospitalization. A total of nine patients (17.0%) experienced postoperative infections, of which six were pulmonary infections, two were intracranial infections, and one was another site of infection. All these infections were alleviated after an intravenous application of antibiotics. Two patients (5.1%) experienced postoperative cerebrospinal fluid leakage, which gradually improved after lumbar drainage. Otherwise, there was no severe postoperative complication such as intracranial hemorrhage, hydrocephalus, meningococcal bulging, and pulsatile exophthalmos.

### Prognosis

**Figure 1:**
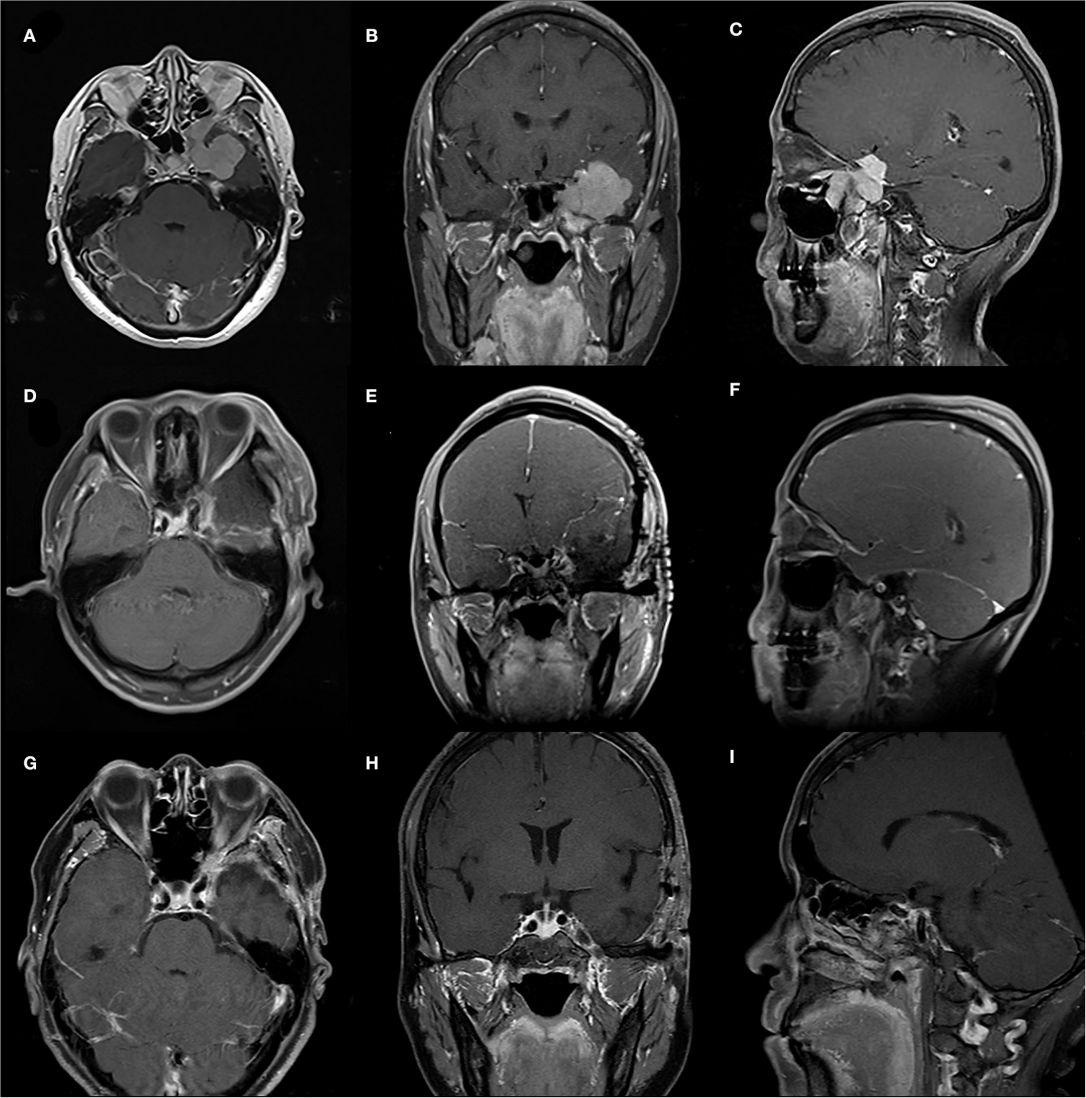
A case of 55-year-old female with a WHO-grade-I meningioma. **(A–C)** Preoperative sellar region MRI showed an irregular lesion of left infraorbital fissure, orbital apex, pterygopalatine fossa, cavernous sinus and middle fossa. **(D–F)** Early postoperative MRI showing gross total resection of the meningioma. **(G–I)** MRI follow-up 6 months postoperatively showing no tumor progression.

**Figure 2:**
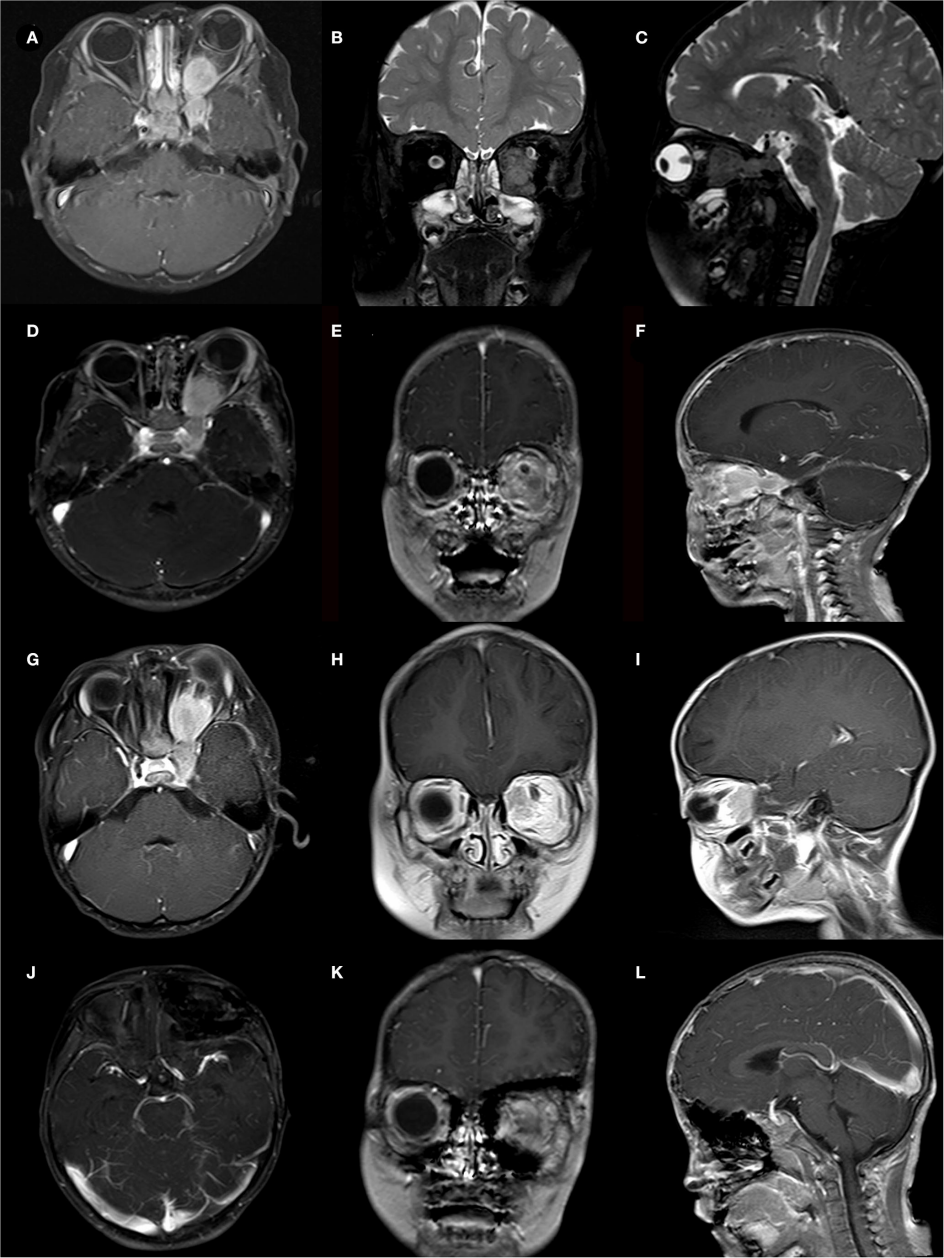
A case of 2-year-old boy presented with a cranial-orbital lesion. **(A–C)** Preoperative MRI showed a left dumbbell-shaped lesion with invasion of extraocular muscles and cavernous sinus. **(D–F)** STR was conducted to remove lesions about 1 cm^*^1 cm from the orbital apex, and pathological biopsy reveals a WHO-grade-I meningioma. **(G–I)** MRI follow-up at 12 months showed tumor progression and the second surgery was taken. **(J–L)** MRI follow-up at 18 months after the second surgery showed no tumor recurrence but the patient suffered severe visual failures.

Excluding ten patients (18.9%) who were lost to follow-up, the follow-up time of the remaining cases ranged from 10 months to 147 months, with an average of 61.5 months. Fifteen patients experienced tumor recurrence with a recurrence rate of 34.9%. For these recurring tumors, seven patients opted for re-operation, two patients underwent radiotherapy, and six patients chose conservative treatment. Two patients experienced multiple recurrences (Case 3,11), and one patient died because of the tumor recurrence (Case 43). Detailed available follow-up data were presented in Table 4. Besides, two representative cases were presented in Figures 1, 2, respectively.

**Table 4:** Follow-up data of the 43 of 53 patients with skull base communicative meningiomas.

**No**	**Sex**	**Age**	**Histopathology**			**Communication**	**EOR**	**Adjuvant**	**Recurrence**	**DFS**	**KPS**
			**WHO grade**	**Ki-67**	**PR**	**way**		**Radiotherapy**	**/Method**	**(month)**	**score**
1	F	49	1	3	/	NC	STR	No	Yes/–	73	70
2	F	40	1	/	/	DB	STR	No	Yes/Surgery	110	90
3	F	51	1	/	/	DB	STR	Yes	Yes/RT	31	70
4	F	46	1	/	+	DB	STR	No	Yes/Surgery	73	40
5	M	58	1	/	/	NC	STR	No	No	124	100
6	M	2	1	2–4	+	NC	STR	No	Yes/Surgery	11	40
7	F	51	1	1	–	NC	GTR	No	No	58	70
8	F	67	1	/	/	DB	STR	Yes	No	107	80
9	F	36	1	/	/	DB	GTR	No	No	103	60
10	F	72	2	5	+	DB	GTR	Yes	No	69	80
11	F	68	1	/	/	DB	STR	No	Yes/Surgery	11	40
12	F	58	1	/	/	DB	GTR	No	No	24	90
13	F	54	1	/	/	NC	STR	Yes	No	35	40
14	F	35	1	/	/	NC	STR	Yes	No	40	80
15	M	45	1	/	/	NC	GTR	No	No	31	100
16	F	49	1	/	/	NC	GTR	No	No	57	40
17	F	43	1	1	+	NC	STR	Yes	No	43	70
18	F	63	1	/	–	NC	STR	No	Yes/–	2	500
19	M	41	2	5	+	NC	GTR	No	Yes/–	11	40
20	F	65	1	3	+	DB	STR	Yes	No	35	100
21	F	13	1	5	+	NC	STR	Yes	No	35	70
22	F	48	1	/	/	NC	GTR	No	No	33	90
23	F	60	1	4	+	DB	STR	No	Yes/Surgery	5	90
24	F	46	2	/	/	NC	GTR	No	Yes/Surgery	17	90
25	M	55	1	/	/	DB	STR	No	Yes/–	3	40
26	F	55	1	20	+	NC	GTR	No	No	12	90
27	M	37	1	/	/	DB	STR	Yes	No	107	90
28	F	42	1	5	+	DB	STR	Yes	Yes/RT	105	90
29	F	51	1	/	/	DB	GTR	No	No	10	100
30	F	71	1	3	+	DB	GTR	No	No	43	60
31	F	53	1	/	/	NC	GTR	No	No	22	90
32	F	50	1	/	/	NC	GTR	No	No	19	100
33	F	44	1	3	+	NC	GTR	No	No	17	90
34	M	52	1	/	/	NC	GTR	No	No	19	100
35	F	51	1	/	/	NC	GTR	No	No	70	50
36	F	50	2	/	/	DB	GTR	Yes	No	62	40
37	F	60	1	1	+	DB	STR	No	Yes/–	4	40
38	M	47	2	/	/	NC	STR	No	Yes/Surgery	20	90
39	F	31	1	/	/	NC	GTR	No	No	54	40
40	M	58	1	/	/	DB	GTR	No	No	48	100
41	F	59	2	/	+	DB	GTR	Yes	No	38	90
42	M	47	1	/	/	DB	GTR	No	No	33	70
43	F	7	2	<1	+	DB	STR	No	Yes/–	3	DEAD

*EOR, extension of resection; NC, natural cavity; DB, destruction of bone; RT, radiotherapy; PR, progesterone receptors*.

After excluding one dead patient, the average KPS score of the remaining patients at the last follow-up was 73 (ranging from 40 to 100). Preoperative exophthalmos in 16 patients was successfully alleviated by the surgery, but one patient without preoperative exophthalmos suffered new-onset postoperative exophthalmos. In 31 patients with preoperative visual failures, the vision of 18 patients (58.1%) was improved, though the postoperative visual acuity of two patients was lower than before the operation. The disorder of ocular movement improved in four out of six patients (66.7%). Besides, four patients suffered postoperative ptosis.

**Figure 3:**
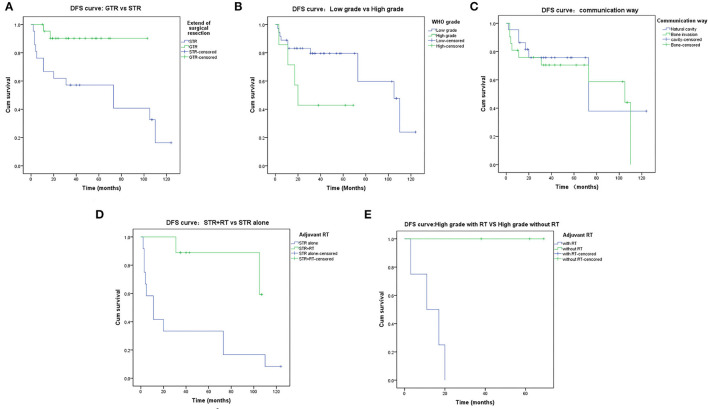
Disease-Free-Survival rates. **(A)** DFS of the extent of resection: GTR vs STR. **(B)** DFS of the WHO grade: Low grade vs. High grade. **(C)** DFS of the communication way: Natural cavity vs. Destruction of Bone. **(D)** DFS of the STR: STR+RT vs. STR alone. **(E)** DFS of the high-grade meningiomas: with adjuvant RT vs. without adjuvant RT.

The 5-year DFS rate was 73.1%. Some possible prognostic factors were analyzed, including the EOR (*p* = 0.013, Figure 3A), the histological grade (*p* = 0.051, Figure 3B), and communication way (*p* = 0.600, Figure 3C). The EOR showed a significant statistical relationship to DFS by log-rank analysis. In addition, adjuvant radiotherapy proved beneficial in patients with subtotal resection (*p* = 0.010, Figure 3D) and in patients with high-grade meningiomas (*p* = 0.018, Figure 3E). Age, sex, the extent of surgical excision, the histological grade, and the communication way were recorded and analyzed by multivariate analysis. The result showed that the EOR (*p* = 0.009) and the histological grade (*p* = 0.007) were significantly related to the prognosis. Detailed multivariate analysis data are presented in Table 5.

**Table 5:** Factors associated with DFS in 43 patients with skull base meningiomas with extracranial extensions.

**Variables**	**Uni-variate**	**Multivariate**	
	***P*-value**	***P*-value**	**HR (95% CI)**
Age (<50)	0.954	0.756	/
Sex	0.627	0.588	/
Histological grade	0.051	0.007	8.423 (1.778–39.905)
Extent of resection	0.013	0.009	0.084 (0.013–0.536)
Communication way	0.600	0.809	/

## Discussions

### Incidence, Age, Sex, and Histopathology

The previous literature rarely reported on the series of skull-base communicative meningiomas (4–9). Among all meningiomas treated at our institution from 2009 to 2020, the incidence of skull-base meningiomas with excranial extensions was only 0.74%, confirming its rarity. The average age of our series was 45.9 years old, lower than the 47.9–64 years old reported in the previous series (4–7, 10). Such an age distribution may be correlated with the origin of our data from the patients with initial meningiomas, whose mean age was usually less than the age of the relapsed case. Similarly, Meling et al. (11) claimed that many skull-base meningiomas series had lower median ages than non-skull-base meningiomas cohorts, and most of the old patients had more likely non-skull-base meningiomas. As far as the sex ratio is concerned, the ratio of men to women in our series was 1:3.1, which was consistent with the women predominance of meningiomas reported in previous studies (12, 13). Poon et al. (14) found that women were an independent predictor for postoperative complications. However, in our study, gender was not found to be related to prognosis. According to the Central Brain Tumor Registry of the United States (CBTRUS), the histological grade was based on the WHO classification with overall proportions of WHO I, II (atypical), and III (anaplastic) intracranial meningiomas of 81.1, 16.9, and 1.7%, respectively (13). In addition, skull-base meningiomas had been found a lower risk of high-grade meningiomas (WHO II or III) (11, 15). In our series, 84.9% of meningiomas were WHO grade I meningiomas, which was consistent with the rules reported in the previous literature. Our series did not find grade III meningiomas.

### Communication Ways and Clinical Symptoms

Meningiomas can communicate through the anterior, middle, and posterior cranial base, invading many important structures, such as orbits, nasal cavity, anterior or middle cranial fossa, pterygopalatine fossa, infratemporal fossa, and neck. The orbit is the most common site of extracranial extension by skull-base meningiomas. The cranial cavity and the orbit are connected through the optic foramen and the supraorbital fissure. Meningiomas that occur in the cranial cavity or the orbit can enter the orbit or the cranial cavity through the optic foramen, supraorbital fissure, or the bone between them, forming cranio-orbital communicative lesions (16). The cranio-orbital junction area is mainly where the cranial nerves II, III, IV, and VI exit the skull. At the same time, the orbital volume is fixed, and the content structure is complicated. Intraorbital lesions often cause an increase in intraorbital pressure. Therefore, cranio-orbital communication lesions are mainly manifested as exophthalmos, eye movement disorders, visual field disorders, diplopia, and so on, which is consistent with previous reports in the literature (2, 9, 17). In this group of cases, 26 patients have exophthalmos as the primary manifestation, accounting for 49.1%, which recommends that those with exophthalmos should actively undergo a radiological examination to exclude the possibility of cranio-orbital communicative lesions. Communicative lesions invade the nasal cavity and the olfactory groove may cause dysosmia, and three patients suffered from olfactory disorders in our series. It is worth mentioning that meningiomas communicating with the posterior skull base are really rare. In recent literature, meningiomas with cervical extension occur in 0–1.4% of all cases of intracranial meningiomas (18–20). In the study of communicative meningiomas, meningiomas communicating through the neck or parapharyngeal space account for only 0–8.8% (4, 5). Correspondingly, no posterior skull-base communication was included in our series.

### Radiological Findings

In our series, most communicative meningiomas presented irregular shapes and clear tumor-brain interfaces. Patients with skull-base communicative meningioma often spread and grow through the anatomical orifices of the skull base. Due to the limitations of the orifices, the shape is mostly irregular dumbbell-shaped (21, 22). In addition, in the MRI enhancement phase, most of the lesions were heterogeneous enhancement. The heterogeneous enhancement may be caused by the presence of necrosis, calcification, and cystic degeneration. Because of fast proliferation, the central area of the tumor often lacks blood circulation, leading to avascular necrosis or cystic degeneration (23). Therefore, it needs to be distinguished from glioma and other intra-axial tumors. Skull invasion is an important feature of communicative meningiomas. A total of 55.3% of the tumors in this series have invaded the skull, including hyperostosis and destructive absorption of the cranial bone, which was equivalent to the incidence reported in the previous literature (24–26). Interestingly, peritumoral edema is common, and the occurrence rate of peritumoral edema in our group is as high as 36.8%. Peritumoral edema comes from vasogenic brain edema or cerebral gliosis because of chronic brain stress and other factors (27). Conditionally, skull-base communicative meningiomas also preserve some of the imaging features of meningiomas, such as a dural tail sign or dural enhancement. The dural tail sign is correlated with tumor dural infiltration and reactive angiogenesis, draining into the adjacent dura (28). In conclusion, the imaging characteristics of skull-base communicative meningiomas could be summarized as follows: heterogeneous enhancement, high bone invasion rate, high incidence of peritumoral edema, and high dural tail sign rate.

### Surgical Strategies and Outcome

The best choice for the treatment of skull-base communicative meningiomas is surgery, whose purpose is to remove the tumor, relieve intraorbital pressure, and save vision, and at the same time clarify the pathology to guide further treatment. Since the anterior, middle skull-base area has important structures such as the optic foramen, superior orbital fissure, and cavernous sinus, it is one of the areas with the most nerves passing through the skull base and the most complex structure, making the operation of skull-base communicative lesions difficult and more complications (29). Communicative lesions are often treated by ophthalmologists and neurosurgeons separately. Ophthalmologists usually remove intraorbital tumors *via* an orbital approach. (30) This surgical method cannot expose the tumor well, and the surgical field of view is limited. In addition, it is easy to damage the intraorbital and intracranial structures such as nerves and blood vessels, leading to a high complication rate (31). With the advancement of skull-based microneurosurgery technology, at present, skull-base communicative tumors are mostly resected by the transcranial approach. The most commonly used surgical approaches are the orbitofrontal approach, pterional approach, and orbital zygomatic approach (32–35). The orbitofrontal approach can expand the exposure of the skull base and orbital contents, and the surgical exposure space is broad, reducing the probability of damage to the orbital tissue. At the same time, fully opening the superior orbital wall can significantly relieve the intraorbital pressure and play a good decompression effect, suitable for anterior cranial fossa base, medial orbital wall, and superior orbital wall lesions. The pterional approach can fully abrade the sphenoid ridge, anterior clinoid process, orbital roof lateral wall, etc., exposing the superior orbital fissure, foramina opticum, etc., suitable for lesions located in the lateral orbit, sella area, and middle cranial fossa. The orbital zygomatic approach involves resection of the frontotemporal bone, superior orbital wall, lateral orbital wall, and zygomatic arch. It is conducive to temporal muscle retraction and exposure Infratemporal fossa, which has a wide exposure range, suitable for tumors involving the cavernous sinus, infratemporal fossa, and interpeduncular cistern (36). In addition, the endoscopic endonasal approach can be used for skull-base tumors located in the midline and extending to the nasal cavity and paranasal sinuses. The endoscopic endonasal approach has the advantages of no external incision, reduced brain retraction, and direct contact with midline skull-base lesions (9, 37).

Our series selected the surgical approach based on the path of communication of the lesion and the location of the subject of the tumor by preoperative imaging examination. The orbitofrontal approach was mainly used for the tumor subject in the orbit and the anterior cranial fossa. When tumors were communicated mainly through the optic nerve foramen or supraorbital fissure, or located in the posterior orbit and sellar area or the middle cranial fossa, pterional approach would be preferred. If the lesion was located in the midline and extended to the nasal cavity and paranasal sinuses, the endoscopic endonasal approach was the best choice. Eventually, we achieved 62.3% of the GTR of the meningiomas by our surgical approach selection. At the same time, the patients with large residual cavities after surgery were filled with autologous fat tissue, and the skull base was reconstructed. Postoperative complications such as cerebrospinal fluid leakage, intracranial hemorrhage, meningoencephalopathy, and pulsatile exophthalmos were rare, and there was no perioperative death. Most of the postoperative complications were infections, which may be related to the difficulty of the resection of the communicative lesions, longer operative time, and longer postoperative bed rest. The presence of cerebrospinal fluid leaks was associated with severe bone destruction of the skull base, but both were corrected by continued postoperative lumbar drainage. In addition, the clinical symptoms of most patients were significantly improved after surgery.

### Prognosis

In our cohort, the overall total recurrence rate was 34.9%, which was in accordance with the previous reports in the literature (7–46.4%) (4–6, 37–39). The most concerning prognostic factors included the EOR, the histological grade, and adjuvant radiotherapy after surgery.

Among these factors affecting prognosis, the EOR is the most critical (4–6, 40). In our study, log-rank analysis and the Cox proportional hazards model were conducted to test the relationship between the EOR and the recurrence of meningiomas. The results confirmed that GTR could significantly reduce the recurrence rate (*P* = 0.013 in log-rank analysis and *P* = 0.009 in multivariate analysis). It is worth noting that some scholars pointed out that the surgical aim should be the relief of leading symptoms rather than radical resection (33). Recurrence occurred respectively in 9.1% of cases with GTR and 61.9% of cases with STR. Considering the low recurrence rate by GTR, we believed that the first resection should be as complete as possible.

About histological grades, associations between histological grades and recurrence in communicative meningiomas have been summarized in previous research, with recurrence rates of 21.1, 58.5, and 50.0% for WHO grades I, II, and III (4). Correspondingly, recurrence was seen respectively in 30.6% of patients with WHO grades I meningiomas and 57.1% of patients with WHO grades II meningiomas in our cohort. Histological grades were also significantly related to DFS by multivariate analysis (*P* = 0.007). Besides WHO grades, the Ki-67 index is a valuable marker to predict meningioma recurrence, especially in histologically borderline meningiomas and possibly to identify the type of low grade of meningiomas at risk of recurrence (4, 38). Besides, the insufficiency of PRs is associated with prognosis and is an important factor in recurrence and survival (41). Regretfully, we did not find an association between the Ki-67 index, PR and tumor recurrence, which may be related to the lack of further pathological data in our series.

Under what circumstances adjuvant radiotherapy is required to be conducted is still controversial in previous studies. Most studies pointed out that patients with high-grade meningiomas and subtotal resection were beneficial after adjuvant radiotherapy (39, 42). On the contrary, some authors claimed that WHO grade-II meningiomas need to be based on conditions such as KI-67 to make a decision on whether to adjuvant radiotherapy or not (4, 43). Our point of view is that in the case of communicative meningiomas, incomplete resection combined with adjuvant radiotherapy is safer and more beneficial for patients when it is challenging to achieve GTR and in the case of high-grade meningiomas. Adjuvant radiotherapy was conducted in 13 patients with subtotal resection or WHO grade-II meningiomas in our cohort, and only two patients suffered tumor recurrence, which demonstrated that adjuvant radiotherapy could improve the prognosis of patients.

### Limitations

Our research has the following limitations. First of all, the study was conducted retrospectively, which resulted in some inherent biases. Second, the sample size needs to be expanded and the follow-up times are required to be extended to acquire more clinical characteristics and prognostic information. Third, some sections of imaging were unavailable in our PACS system. This is caused by part of patients who underwent scans of specific parts like the saddle area or had preoperative imaging examinations in other hospitals. Finally, some patients refused further immunohistochemical analysis after confirming the pathological diagnosis of meningioma, which resulted in a lack of further immunohistochemical analysis.

## Conclusions

Skull-base meningioma with extracranial extension is a rare type and presents a female predominance. Compared with intracranial meningiomas, patients with communicative meningiomas are much younger and show a higher tendency to develop low-grade tumors. Typical imaging features include heterogeneous enhancement, high bone invasion rate, high incidence of peritumoral edema, and high dural tail sign rate. According to the tumor location and communication way showed by the preoperative imaging, routine craniotomy or endoscopic endonasal approach can be adopted to achieve the maximum tumor resection. Reasonable postoperative skull-base reconstruction strategies can minimize postoperative complications. Low histological grade, GTR, and adjuvant radiotherapy often indicate a good prognosis.

## Data Availability Statement

The raw data supporting the conclusions of this article will be made available by the authors, without undue reservation.

## Ethics Statement

The studies involving human participants were reviewed and approved by West-China Hospital Research Ethics Committee. Written informed consent to participate in this study was provided by the participants' legal guardian/next of kin.

## Author Contributions

JX, WH, and ZL: conception and design. WH, DJ, HT, and LT: acquisition of data. WH, DJ, and LT: analysis and interpretation of data. WH: drafting the article. ZL: critically revising the article. JX: reviewed submitted version of manuscript and approved the final version of the manuscript on behalf of all authors. All authors contributed to the article and approved the submitted version.

## Funding

This work was supported by key research and development project of the science and technology department of Sichuan Province (Grant No. 2019YFS0392/3) and 1.3.5 project for disciplines of excellence, West China Hospital, Sichuan University (Grant No. ZYJC18007).

## Conflict of Interest

The authors declare that the research was conducted in the absence of any commercial or financial relationships that could be construed as a potential conflict of interest.

## Publisher's Note

All claims expressed in this article are solely those of the authors and do not necessarily represent those of their affiliated organizations, or those of the publisher, the editors and the reviewers. Any product that may be evaluated in this article, or claim that may be made by its manufacturer, is not guaranteed or endorsed by the publisher.
